# Another Rubber Suit

**Published:** 1868-11

**Authors:** 


*Another Rubber Suit.*—Goodyear *vs* Newbrough, before Judge Blatchford.

"This was a motion for an injunction against Dr. Newbrough, the inventor of the so-called idiozed hard rubber.

Plaintiffs claim that their patent includes all hard rubber, whether made strictly in accordance with the terms of their patent or not. That the hard rubber made by the defendant is merely a colorable difference.

The main point relied upon by them was that the defendant required sulphur in his process, that the hardening process was due to the presence of this sulphur, and not to the iodine.

Yesterday the defendant introduced affidavits from Professor Silliman, of Yale College; Professor Chandler; of Columbia College; Professor Seeley, Engelhardt, and Wurtz, of New York, and Professor Torrey, the Assayist of the U. S. Assay Office, showing that the hardening was due to the action of iodine alone. The more fully to corroborate this, they attached nearly a hundred specimens of the rubber, in all forms and colors, made by themselves, and made without any sulphur.

The complainants, pretending a surprise upon this, demanded that the whole case should go before a chemical expert to decide upon the facts alleged. The defendant resisted this motion as being made merely for delay, and wholly unprecedented.

The judge refused the reference, stating that he never heard of such a motion being granted in this country, and that furthermore, the defendant had already procured *all* the first-class chemists in the city, and it would be unfair to them to have their opinions criticised, as they would be if he chose one who had not already expressed an opinion.

The complainants then withdrew their motion for an injunction, the defendant protesting against it, and claiming that he had a right to a hearing, having incurred so much expense in the preparation of the case.

The court decided that there was no power to prevent them from withdrawing their motion.

On defendant's motion, it was ordered that the complainant proceed immediately to take testimony for final hearing. The defendants then requested leave to have their affidavits and exhibits sent to such circuit courts as they may desire, to resist other motions, and more particularly to apply to set aside an injunction, granted *ex parte*, against one of defendant's licensees in Pittsburg, which the court ordered.

The case in effect tests the question whether the Goodyear monopoly can crush out *all* inventions, however made, and whether a *man can be found* with money and *pluck* to fight them to the Supreme Court.

Charles F. Blake and Mr. Pollock, counsel for complainants.

John A. Foster and S. D. Law, counsel for defendants."

